# The Vermiform Appendix and Its Pathologies

**DOI:** 10.3390/cancers15153872

**Published:** 2023-07-29

**Authors:** Marian Constantin, Livia Petrescu, Cristina Mătanie, Corneliu Ovidiu Vrancianu, Adelina-Gabriela Niculescu, Octavian Andronic, Alexandra Bolocan

**Affiliations:** 1 Institute of Biology of Romanian Academy, 060031 Bucharest, Romania; cvgmarian@gmail.com; 2 The Research Institute of the University of Bucharest, ICUB, 050095 Bucharest, Romania; adelina.niculescu@upb.ro; 3 Department of Anatomy, Animal Physiology and Biophysics, DAFAB, Faculty of Biology, University of Bucharest, 050095 Bucharest, Romania; livia.petrescu@bio.unibuc.ro (L.P.); cristina.matanie@bio.unibuc.ro (C.M.); 4 Microbiology—Immunology Department, Faculty of Biology, University of Bucharest, 050095 Bucharest, Romania; 5 National Institute of Research and Development for Biological Sciences, 296 Splaiul Independentei, District 6, 060031 Bucharest, Romania; 6 Department of Science and Engineering of Oxide Materials and Nanomaterials, Politehnica University of Bucharest, 011061 Bucharest, Romania; 7 University Emergency Hospital, Carol Davila University of Medicine and Pharmacy, 020021 Bucharest, Romania; andronicoctavian@gmail.com (O.A.); alexandra.bolocan@umfcd.ro (A.B.)

**Keywords:** vermiform appendix, appendicitis, appendiceal tumors, adenocarcinoma

## Abstract

**Simple Summary:**

Despite its small size, the vermiform appendix is an organ with several physiological roles and various pathologies, the most common of which is acute appendicitis. The other pathologies of the vermiform appendix, especially its neoplasia are rare and often go unnoticed and are accidentally identified during appendectomies performed for other reasons. In the early stages, most appendiceal neoplasms are not detected; however, in the advanced stages, they may mimic the symptoms of acute appendicitis. In addition, due to massive mucus production, mucinous neoplasms, especially adenocarcinomas, may fistulize into adjacent structures, some identified after perforated organ pathology. The general treatment for appendiceal pathologies, including neoplasms, is complete surgical excision of the appendix, with or without the right hemicolectomy. Life expectancy is somewhat longer for low-grade mucinous tumors and peaks for well-differentiated, small, metastasis-free neuroendocrine tumors of the appendix occurring in children.

**Abstract:**

The vermiform appendix is a muscular cylindrical structure originating near the junction of the cecum and ileum, averaging 9 cm (5–35 cm) in size. As the most mobile viscera, it can adopt several positions, the most common being the retrocecal position. Perceived as an atavistic organ lacking physiological relevance, the vermiform appendix appears to be involved in immune function, serving in the maturation of B lymphocytes and the production of immunoglobulin A, in endocrine function, excreting amines and hormones in the 2–3 mL of mucus secreted daily, and in digestive function, by storing beneficial bacteria from where they can recolonize the colon. With a lumen of about 6 mm, the vermiform appendix has a reduced storage capacity, so any blockage of the appendix with fecoliths (fecaliths), seeds derailed from the colon, or enlarged lymph nodes prevents drainage and intraluminal accumulation of secreted mucus. Unable to relax, the appendix wall severely limits its intraluminal volume, so mucus accumulation leads to inflammation of the appendix, known generically as appendicitis. In addition, the vermiform appendix may be the site of the development of neoplastic processes, which may or may not involve mucus production, some of which can significantly affect the standard of living and ultimately lead to death. In general, mucinous tumors may have a better prognosis than non-mucinous tumors. This review takes a comprehensive path, starting by describing the anatomy and embryology of the vermiform appendix and further detailing its inflammatory pathologies, pathologies related to congenital anomalies, and appendix tumors, thus creating an up-to-date framework for better understanding, diagnosis, and treatment of these health problems.

## 1. Introduction

The vermiform appendix has more intense physiological activity in childhood when immune functions seem very active, after which it seems to restrict its role in the body significantly [1]. However, throughout life, it can be the site of various pathologies, of which only acute appendicitis has specific symptoms (abdominal pain, descending to the lower right quadrant, nausea, vomiting, and anorexia), and the rest of the pathologies have similar symptoms or lack specific symptoms [2,3]. Pathologies of the appendix are categorized into inflammatory pathologies (acute appendicitis), pathologies related to congenital anomalies of the appendix and other related diseases, and tumors of the appendix [4,5].

Acute appendicitis is by far the most common pathology of the appendix and one of the most frequent causes of acute abdominal surgery, as most cases, especially those developing appendicoliths (fecoliths), require urgent removal of the inflamed appendix. With a poorly expandable wall that may become ruptured, the inflamed appendix can release its contents into the abdominal cavity, resulting in peritonitis [3,6,7]. Pathologies related to congenital anomalies include appendicular agenesis (complete absence of the vermiform appendix), appendix duplications (existence of more than one appendix, partially or integrally), appendiceal diverticulosis (occurrence of one or more appendiceal diverticula), volvulus of the appendix (abnormal twisting of the appendix on itself), location of the appendix in an unusual position due to its malrotation or situs inversus, hypoplasia of the appendix, or appendix fistulizing into the umbilicus [8].

Although appendiceal tumors occur very rarely, they are of epithelial origin and are classified by the World Health Organization as hyperplastic polyps, sessile serrated lesions without dysplasia, serrated dysplasia (low or high grade), appendiceal mucinous neoplasms (low or high grade), appendiceal adenocarcinomas NOS (not otherwise specified) (mucinous and signet-ring cell), undifferentiated carcinomas NOS, goblet cell adenocarcinoma, and appendiceal neuroendocrine neoplasms (well-differentiated neuroendocrine tumors: neuroendocrine tumors NOS, neuroendocrine tumor, grades 1–3, L-cell tumor, glucagon-like peptide-producing tumor, PP (pancreatic polypeptide)/PYY (peptide YY)-producing tumor, enterochromaffin-cell carcinoid, and serotonin-producing carcinoid; poorly differentiated neuroendocrine carcinomas or neuroendocrine carcinoma NOS, with large or small cells, and mixed neuroendocrine-non-neuroendocrine neoplasms) [9,10].

In the following sections, this paper aims to review the anatomy, embryology, and pathologies of the vermiform appendix, detailing the types of tumors and indicating, where possible, their main genetic alterations.

## 2. The Vermiform Appendix—Anatomy and Embryology

The vermiform appendix is a cylindrical muscular structure attached to the large intestine, originating near the ileocecal valve from the cecal fundus or the posteromedial border of the cecum, approximately 2 cm from the junction of the cecum with the ileum. It is worm-shaped, hence the name vermiform appendix, and can range in length from 5 to 35 cm, on average 9 cm [11]. Although it is considered an atavistic organ and of no importance to the body, the vermiform appendix appears to have immunoprotective and lymphatic functions, especially in childhood; it is involved in the maturation of B lymphocytes and the production of immunoglobulin A, a storage function for useful bacteria present in the colon, and an endocrine function, producing amines and hormones, which are released in the 2–3 mL of mucus it secretes each day [11,12].

**Figure 1:**
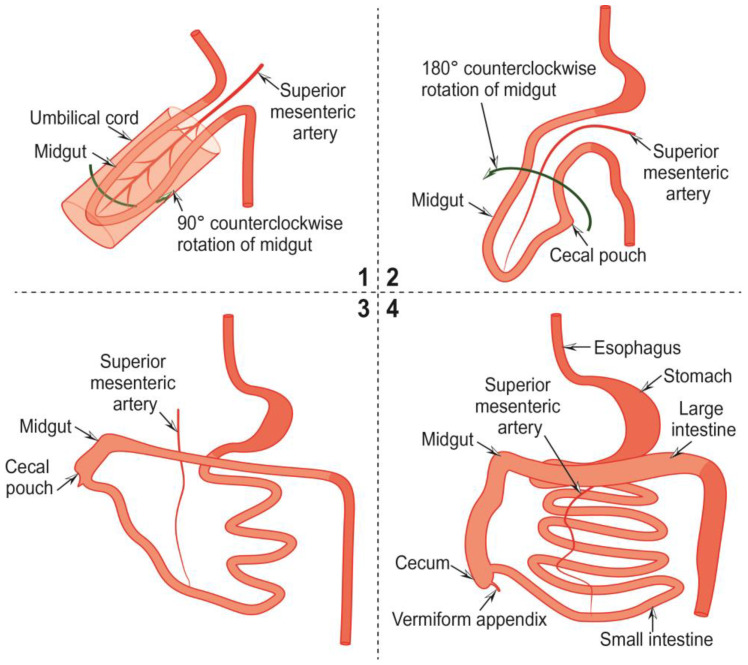
Stages of embryonic development of the vermiform appendix. (**1**). At about 4 weeks; (**2**). In the fifth week; (**3**). At about 12 weeks; (**4**). In the final stage.

The development of the vermiform appendix is closely related to that of the midgut and begins in the fifth week of embryonic development. In the fourth week, the midgut, supplied by the superior mesenteric artery, herniates into the umbilical cord. At the same time, the upper and lower segments of the intestine are held in place by retention bands (Figure 1 (1)) [13].

In the fifth week, with counterclockwise rotation of the intestine, the middle segment of it returns to the abdomen, and histological differentiation of the constituent parts begins (Figure 1 (1,2)). The appendix becomes histologically visible within the cecum three weeks later. The cecum ascends into the upper abdomen by reducing the post-arterial gut segment ar week 12 and rotating the intestine by 270°. There, parts of the primitive mesentery fuse, attaching the ileum and the ascending and descending colon to the posterior abdominal wall and, at the same time, stretching the colon. As the colon stretches, the cecum separates into a cecum pouch (Figure 1 (1–3)), and the vermiform appendix sprouts from it as a small bud. Meanwhile, it is pushed towards the junction of the cecum with the ileum to its final position. The appendix becomes visible in the eighth week of embryonal development (Figure 1 (1–4)) [13].

**Figure 2:**
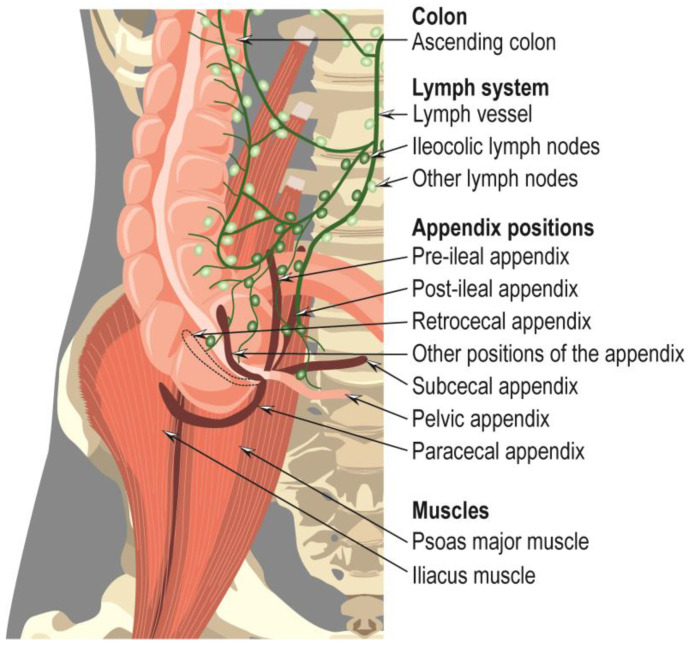
The possible positions of the vermiform appendix, with respect to the ascending colon, main lymphatics, and iliac and psoas muscles.

The appendix is the most mobile viscera and seems to adopt random positions [11,12]: retrocecal/retrocolic, pelvic, post-ileal, subcecal, pre-ileal, paracecal, etc. (Figure 2) [14]. Nonetheless, the retrocecal position is noted to be the most common [12].

Together with the ileum and cecum, the vermiform appendix is vascularized by the appendiceal artery, a branch of the ileocolic artery, which in turn is derived from the superior mesenteric artery. The ileocolic artery has five branches: the ileal branches, the anterior cecal artery, the posterior cecal artery, the colic ramus or ascending ramus, and the appendiceal artery, of various origins. Thus, in 35% of cases, it is derived from the iliac branches, in 28% of cases from the ileocolic artery, in 20% of cases from the anterior cecal artery, in 12% of cases from the posterior cecal artery, in 3% of cases from the ileocecal artery, and in 2% of cases from the colonic branch. The ileocolic and right colic veins, which drain the appendix, accompany the arteries, and drain the lymph via the ileocolic nodes, adjacent to the superior mesenteric artery, and via the celiac nodes, carry it to the cisterna chyli [11,12].

**Figure 3:**
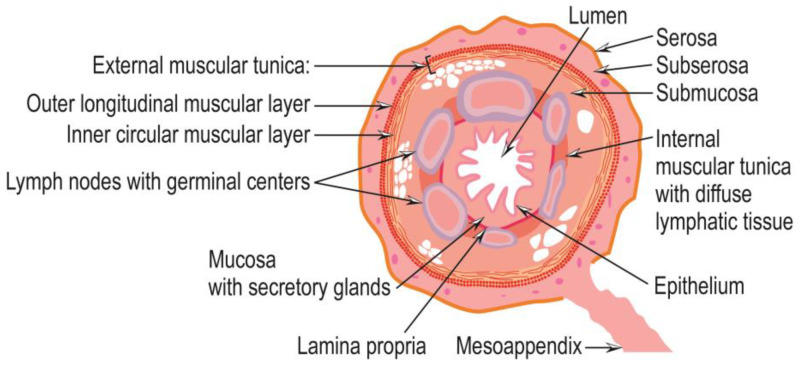
Schematic cross-section of the appendix showing its layered structure.

Histologically, the appendix wall is similar to that of the colon. It contains, from the outside to the appendix lumen, the same components as the colon wall: serosa, subserosa, external muscular tunica, submucosa, a layer containing lymph nodes, an internal muscular layer with diffuse lymphatic tissue, mucosa, lamina propria, and epithelium. The external muscular tunica contains both longitudinal fibers (outer layer) and circular fibers (inner layer). The diffuse lymphatic tissue, which populates the internal muscular layer, is more abundant than its colonic counterpart, indicating a more substantial involvement of the appendix in immune function. The mucosa is the most prominent secretory layer of the appendix, and the epithelium has discrete invaginations, forming crypts separated by their lamina and bearing enteroendocrine cells [11] (Figure 3).

On ultrasound images, the vermiform appendix appears as a tubular structure with a blind end, attached to the cecum, compressible, filled with fecal matter, fluid, or air, and having a transverse diameter of about 6 mm from serosa to serosa. In most images, the five components of the appendix wall appear as alternating layered bands of varying echogenicity [11,15]. Computed tomography images show the appendix as a vermiform structure, approximately 6 mm long, thin-walled, and filled with gas or contrast material. At the same time, the presence of fluid may indicate appendiceal pathology. The presence of periappendiceal adiposity is a sign of inflammation. In several cases, the vermiform appendix cannot be visualized [11,16]. The appendix appears on the nuclear magnetic resonance images as a hyposensitive T1 and T2 structure with a transverse diameter of approximately 6 mm and walls thinner than 2 mm. The presence of periappendiceal adiposity and intra-appendiceal fluid indicates appendiceal pathology [11,17].

Pathologies of the vermiform appendix can be classified into three types: inflammatory pathologies (acute appendicitis), pathologies related to congenital anomalies of the appendix, and other related diseases and tumors of the appendix. These categories are discussed in more detail in the following sections.

## 3. Inflammatory Pathologies of the Vermiform Appendix

Acute appendicitis is the most common inflammatory pathology of the appendix and affects especially young people [11,16] in the second and third decades of life [11,12,18]. In the 1990s, the lifetime occurrence of acute appendicitis was 7% [18], but by 2019, the incidence had increased to 8.7% [19]. Thus, globally, in 2019, 17.70 million new cases of acute appendicitis were reported, with an age-standardized incidence rate of 229.86 per 100,000 [20], up to 20.5% from 1990 [19]. In terms of gender, the lifetime risk of acute appendicitis is 8.6% in women and 6.7% in men [2]. The incidence of acute appendicitis appears to be correlated with increasing ambient temperatures. Thus, for every 5.56 °C increase in temperature, a 1.3% increase in the incidence of acute appendicitis is reported for temperatures less than or equal to 10.56 °C and 2.9% for temperatures greater than 10.56 °C [21]. Children under two years old rarely develop appendicitis due to the inverted pyramid configuration of the appendix, which reduces the risk of obstruction of the appendix lumen [12]. For the first time, the diagnosis of acute appendicitis as a surgical entity was made by Reginald H. Fitz in 1886, who recommended appendicectomy as the only treatment [11].

Appendicitis is recognized by its symptoms: peri-umbilical pain, which migrates to the right lower quadrant, especially external pressure, nausea, and lack of appetite (anorexia). The leading cause of acute appendicitis is the obstruction of the appendix lumen with fecoliths, intestinal worms (especially ascarids), seeds, and barium sulfate ingested for digestive imaging. Sometimes, the same effect results in hypertrophy of lymphoid tissue, which compresses the appendix lumen, but in many cases, acute appendicitis has no obstructive reason. Blocking the communication of the appendix lumen with the cecum prevents drainage of mucus produced by the appendix without affecting the normal mucus secretion of 2–3 mL per day. In a short time, the appendix lumen becomes full. The presence of horizontal collagen fibers in the appendix wall ensures minimal expansion, maintaining an intra-appendicular volume of about 1 mL. Soon after, the lumen is filled with mucus, which exerts pressure on the appendix walls, causing distention. Intraluminal pressure is also amplified by the multiplication of the appendiceal microbiota, which produces toxins [11,22].

Appendiceal distention stimulates nerve endings of the visceral afferent fibers, producing diffuse, dull pain in the mid-abdomen, which is amplified by applying local pressure from outside the abdomen, such as right thigh flexion on the abdomen. Increased intraluminal pressure of the appendix causes occlusion of capillaries and venules in its wall, but not of arterioles, through which blood flow remains constant, leading to vascular congestion. This affects the lining of the appendix and allows for the invasion of microorganisms into the appendix wall. The absorption of microbial toxins and necrosis products into the systemic circulation favors the development of fever, tachycardia, and leukocytosis. The continued distention of the appendix leads to reflex nausea, vomiting, and the amplification of diffuse abdominal pain, which, after the inflammatory process, affects the serosa of the appendix and the nearby parietal peritoneum, migrating to the right lower quadrant. In areas with reduced blood supply, infarcts, necrosis, and perforation of the appendix wall occur, especially in areas affected by infection with microorganisms [11].

In addition, different atypical presentations of appendicitis have been reported, including left-sided abdominal pain localized in the left upper quadrant (in patients with gut malrotation or *situs inversus*), diarrhea (in advanced appendicitis, especially in patients with inter enteric abscesses), severe right hemiscrotal pain (in adult males), genitourinary complaints (in adult females), and strangulated inguinal hernia with non-specific symptoms (in the elderly). Moreover, pregnant patients may present with a series of uncommon symptoms, including gastroesophageal reflux, malaise, pelvic pain, epigastric discomfort, indigestion, flatulence, dysuria, and altered bowel habits [7,23,24]. Thus, diagnosing acute appendicitis is a challenging task in which clinicians must be aware of anatomical variations of the appendix, take into consideration both typical and atypical symptoms, and confirm their suspicions with appropriate imaging studies to provide treatment promptly [25].

Secondarily, appendicitis may be associated with Amyand hernia (with four subtypes: subtype 1, inguinal hernia and normal appendix; subtype 2, inguinal hernia and acute appendicitis without inflammation; subtype 3, inguinal hernia and acute appendicitis with inflammation of the abdominal wall or peritoneum; and subtype 4, inguinal hernia and appendicitis associated or not with abdominal pathology), Garengenot’s femoral hernia or Spigelian hernia [11].

## 4. Pathologies Related to Congenital Anomalies of the Appendix and Other Related Diseases

The congenital anomalies of the appendix are agenesia, hypoplasia, or intramural localization, which often can be confused with agenesia, duplication, or triplication of the appendix, which occur quite rarely; the appearance of one or more mucosal folds covering the opening of the appendix in the cecum is called Gerlach’s valve [11]. They are rare and have non-specific symptoms that make them challenging to diagnose and sometimes mistaken for acute appendicitis.

Appendicular agenesia consists of the complete absence of the vermiform appendix and is diagnosed intraoperatively, with some difficulty, only after all abnormal variants of its origin (funnel-like origin, cecal fundus origin, cecal origin, dorsomedial origin, and origin immediately near the ileal orifice) have been exhausted [12].

Appendix duplications consist of the existence of more than one individual, partial or integral, and are sometimes discovered by the occurrence of acute appendicitis consecutive to appendectomy. Based on the morphology of appendix duplications, in 1941, Theodore R. Waugh classified them into three types: (a) an appendix with a common muscular wall and two lumens; (b) two completely separated appendices with origin in the cecum; (c) a normal appendix and a hypoplastic appendix, possibly of atypical origin [12,26]. Currently, appendix duplications are divided into five types: (a) type A—bifid appendix, with a joint base and two tips; (b) type B1—double appendix, originating on both sides of the ileocecal valve; (c) type B2—one normal appendix and one rudimentary appendix, originating on a cecal tenia; (d) type B3—one normal appendix and one appendix originating on the right colonic flexure; (e) type C—double cecum, each with one appendix. Sparsely, triplication of the appendix may exist, accessory appendix without communication with the cecum, and spiral or helical appendix [12].

Appendicular diverticulosis is a rare condition and consists of the congenital or acquired occurrence of one or more appendiceal diverticula, perceived as rudimentary duplications of the appendix [12,27]. It can be differentiated from appendiceal mucosal prolapse (internal or external pseudodiverticulosis) by hiatuses of the muscular tunica at the entrances and exits of blood vessels, forming multiple pseudodiverticula with diameters of 3–5 mm [12]. Appendicular diverticulosis may mimic acute appendicitis, but is reported in people over 40 years of age and is associated with an increased risk of perforation and formation of appendiceal neoplasms such as carcinoids and mucinous adenomas [27].

The volvulus of the appendix is the abnormal twisting of the appendix on itself, resulting in obstruction of the appendix lumen, with acute appendicitis symptoms and all the consequences listed in its description. Immediate surgical intervention is required to prevent gangrene and infection. On CT images, the volvulus of the appendix appears as a heterogeneous mass with the appearance of an abscess [28].

Usually, the vermiform appendix is located in the right lower abdomen. However, there are rare cases in which it is located in unusual positions, inside the abdomen or even in the thoracic cavity [12,29,30], due to malrotation or *situs inversus*. Sometimes the appendix may be hypoplastic [12], even located intercaecal/intramural [31], at first glance, being considered a missing appendix [11], or the appendix can fistulate into the umbilicus (appendix-umbilicus fistula). In cases of an acute abdomen where no apparent cause can be detected, it is necessary to consider the unusual location of the appendix and identify its position by exploratory surgery, performed open, laparoscopically, or robotic, or by using imaging techniques.

## 5. Tumors of the Appendix

Appendiceal tumors are rare medical pathologies of the gastrointestinal tract that are heterogeneous, of epithelial or non-epithelial origin, and have different malignant potentials. The incidence of appendiceal tumors is about six cases per million people annually. Their prognosis depends on the type and grade of the tumor, and the long-term survival rate is between 10 and 90% [32,33,34]. According to the WHO classification of tumors in the appendix [10], appendiceal neoplasia is classified as an epithelial tumor. When only the appendix is involved, primary appendiceal neoplasms are challenging to diagnose preoperatively because they do not have specific symptoms and are frequently mistaken for acute appendicitis. Even intraoperatively, a definitive diagnosis cannot be made quickly, hampered by the associated inflammation. However, postoperative pathological examination can provide the correct diagnosis when the surgeon suspects malignancy. About 1% of the appendix specimens examined pathologically show neoplastic lesions [34]. The tumors of the appendix have epithelial or mesenchymal origin, and, according to the WHO, they are categorized into several types: (i) hyperplastic polyps, (ii) sessile serrated lesions without dysplasia, (iii) serrated lesions with dysplasia, (iv) appendiceal mucinous neoplasms, (v) adenocarcinomas NOS (not otherwise specified), (vi) undifferentiated carcinoma NOS, (vii) goblet cell adenocarcinomas (GCAs), and (viii) neuroendocrine tumors [9].

### 5.1. Hyperplastic Polyps, Sessile Serrated Lesions without Dysplasia, and Serrated Lesions with Dysplasia

Hyperplastic polyps, sessile serrated lesions without dysplasia, and serrated lesions with dysplasia have a serrated shape of the crypt lumen and appear throughout the appendix in post-inflammatory reparative situations, after an episode of acute appendicitis, in appendiceal diverticulitis, or an interval appendectomy. They are found incidentally in appendectomies, occurring with approximately equal frequency in older men and women in the sixth to eighth decades of life. They do not show specific symptoms but, when significant, can lead to appendicitis and, potentially, appendix rupture. In the ICD-0 system, they have coded 8213/0 serrated dysplasia, low grade, and 8213/2 serrated dysplasia, high grade, hyperplastic polyps, and sessile serrated lesion without dysplasia, and in ICD-11 coding, they are coded 2E92.4Y&DB35.0 of other specified benign neoplasms of the large intestine and hyperplastic polyps of the large intestine. In polyps, no tumor-specific mutations were identified [10,35].

Hyperplastic polyps often morphologically resemble their colorectal counterparts. They have straight, elongated crypts with serrations limited to the luminal side, with many goblet cells or a mixture of goblet cells and columnar cells with smaller mucin vacuoles. Cytological atypia is absent or mild, especially in deep crypts, but without cytological dysplasia. They generally have the appearance of discrete polyps or may circumferentially affect the mucosa. Rare villous growths may occur [10,36,37,38,39].

In sessile serrated lesions without dysplasia, the mucosal crypts become elongated, with extensive serrations and dilation to the base, with unusual shapes, which may take on the appearance of letters L or T and unusual villous growth. Crypts are abnormally proliferated and circumferentially involve the mucosa. Cytological atypia may be mild and may include dystrophic goblet cells. The lumen may contain abundant mucin [10,34,40,41]. The most common mutations occur in the *KRAS* (Kirsten Rat Sarcoma Viral Proto-Oncogene) gene, followed by those in the *BRAF* (B-Raf Proto-Oncogene, Serine/Threonine Kinase) gene and, very rarely, in the *RNF43* (Ring Finger Protein 43) gene [35,37,42].

In serrated lesions with dysplasia, the crypts appear distorted, with constriction and dilatation extending to their base, with low-grade or high-grade dysplasia, often with circumferential mucosal involvement and variable villous growth. The dysplasia may be adenoma-like (villous growth with elongated, hyperchromatic, and pseudostratified nuclei, with frequent mitosis and apoptotic bodies, similar to those in colorectal adenomas), serrated dysplasia (with serrated crypts, lined by cuboidal cells to low columnar cells with large hyperchromatic nuclei, low cytoplasmic mucin, and numerous mitoses), or a serrated adenoma-like dysplasia (with complex serrations and villous growth; villi lined by tall columnar cells with eosinophilic cytoplasm, with elongated and slightly hyperchromatic nuclei, but with less atypia than in conventional adenoma-like dysplasia; villi may have abortive-like crypts along their lateral margins). Each serrated polyp shows several types of dysplasia, sometimes with the dysplastic component sharply demarcated from the non-dysplastic areas [10,36,37,42]. The most common mutations occur in the *KRAS* gene [35].

### 5.2. Appendiceal Mucinous Neoplasms

Appendiceal mucinous neoplasms occur in the appendix, have an unknown etiology, and are characterized by mucinous epithelial proliferation with extracellular mucinous excretion and pushing tumor margins. They occur in adults in the sixth or seventh decades of life, with an approximately equal frequency in men and women. They can be asymptomatic when they are incidentally detected or produce appendicitis-like symptoms, sometimes with appendix perforation. When this is present, progressive abdominal distention, umbilical hernia, or the appearance of a palpable mass is observed on abdominal or pelvic examination. Imaging investigations may reveal fluid or a soft tissue mass within the appendix. Curvilinear calcification of the appendix wall may also occur, which is a defining feature, but is present in only about half of the cases [10]. When perforating the appendix wall, mucinous neoplasms may attach the appendix to neighboring organs (e.g., in the psoas muscle), less commonly arising cutaneously through a fistula. It may be diagnosed as a muscular abscess. Mucocele rupture can lead to pseudomyxoma peritonei (PMP) [43,44], a complication that, if untreated, is fatal [45]. In the ICD-0 coding system, they are referred to as 8480/1 low-grade appendiceal mucinous neoplasm and 8480/2 high-grade appendiceal mucinous neoplasm, and according to ICD-11 coding, they are referred to as 2E92.4Y&XH0EK3 of other specified benign neoplasm of the large intestine and mucinous cystic neoplasm with low-grade dysplasia, and 2E61.Y&XH81P3 carcinoma in situ of other specified digestive organs and mucinous cystic tumor with high-grade dysplasia [10].

According to the Union for International Cancer Control (UICC) staging system, low-grade appendiceal and high-grade appendiceal mucinous neoplasms are limited to the submucosa and muscularis propria, considering tumors in situ (pTis), and the caveat with high-grade neoplasm is staged similarly to invasive appendiceal adenocarcinoma. When extending only to the subserosa, they are staged as pT3; when perforating the serosa and involving the serosa of the appendix, they are staged as pT4a. When mucin and/or epithelial cells leave the appendix and reach the surfaces of the peritoneum, the tumors are included in stage pM1, as stage pM1a, when the mucin is acellular, and stage pM1b, when the mucin contains mucinous epithelial cells [10]. Under these circumstances, the diagnosis of low-grade appendiceal mucinous neoplasm depends on the stage of the neoplasia. Neoplasia limited to the appendix has an excellent prognosis.

In contrast, those with peritoneal dissemination have a variable prognosis, depending on the extent of mucinous production and the possibility of achieving complete cytoreduction (CRS) of macroscopically visible tumors in the abdomen. Thus, the best survival rates are achieved with complete cytoreduction associated with hyperthermic intraperitoneal chemotherapy (HIPEC) [10,37,42,46,47,48,49,50]. Because high-grade appendiceal mucinous neoplasia rarely occurs, data on its natural history are scarce. Therefore, it is considered a type of neoplasia with a reserved prognosis, and its treatment consists of cytoreductive surgery followed by hyperthermic intraperitoneal chemotherapy and adjuvant systemic chemotherapy [51]. Following dissemination into the peritoneal cavity, the high-grade appendiceal mucinous neoplasm is assumed to behave similarly to other mucinous tumors with peritoneal spread [10].

Low-grade appendiceal mucinous neoplasm (LAMN) is a unique histological subtype of mucinous neoplasia of the appendix, which is characterized by the replacement of normal mucosal tissue by villous filiform mucinous epithelial proliferations. These partially or entirely invade the appendix wall structures and may cause the appendix to rupture up to the surface of the peritoneum. Wall invasion is confluent, cribriform, destructive, with desmoplasia, and is evidenced by infiltrative growth, which categorizes neoplasia as adenocarcinoma and/or tumor cells floating in the extracellular mucin. The glandular epithelium in the wall is prominent and rounded in shape. Serosa involvement results in mucin on the surface or the replacement of a portion of the hyalinizing wall by strips of low-grade mucinous epithelial cells, which abundantly produce extracellular mucin. Some tumors have cells with large vacuoles filled with mucin that tends to compress their nucleus. Others have an attenuated or flattened appearance of a monolayer of mucinous epithelium. In contrast, others have a wavy or scalloped appearance with columnar epithelial cells and nuclear pseudostratification growing in the fibrotic submucosa. The degree of atypia is low, the appendix wall may be fibrotic, hyalinized, or calcified, and lymphoid tissue is absent [10,37,43,47,52]. Most low-grade appendiceal mucinous neoplasms carry mutations in the *KRAS* gene. Some are associated with mutations in the *TP53* (Tumor Protein 53) *IHC* (immunohistochemistry) gene, and rarely with mutations in the *PIK3CA* (Phosphatidylinositol-4,5-Bisphosphate 3-Kinase Catalytic Subunit Alpha) gene. In many cases, mutations of the *GNAS* (G Protein Subunit Alpha S) gene are present (p.R201H, c.602G>A and p.R201C, c.602C>T), probably with a role in abundant mucin production [53], and in a smaller number of cases; mutations in *FAT4* (FAT Atypical Cadherin 4), *SMAD2* (Mothers Against Decapentaplegic Homolog 2), *AKT1* (AKT Serine/Threonine Kinase 1), *MET* (MET Proto-Oncogene, Receptor Tyrosine Kinase), *JAK3* (Janus Kinase 3), *PIK3CA*, *STK11* (Serine/Threonine Kinase 11), *RNF43*, *APC* (Adenomatosis Polyposis Coli Tumor Suppressor), and *RB1* (Retinoblastoma-Associated Protein) genes, or the coexistence of mutations in *BRAF* and *TP53 IHC* genes or *TP53 IHC* and *RNF43* genes have been reported [10,35,54]. These mutations generally consist of C>T transitions, suggesting that 5-methylcellulose is a possible mutagenic mechanism in these tumors [10,55,56].

High-grade appendiceal mucinous neoplasms (HAMN) are rare and histologically similar to low-grade appendiceal mucinous neoplasms (subepithelial fibrosis, a wide pushing margin, appendiceal rupture, and peritoneal dissemination, with the possibility of peritoneal pseudomyxoma formation). However, the epithelium acquires features of high-grade cellular atypia, with micropapillae and a cribriform or crowded appearance, although the epithelial cells are often arranged in a single layer. They have large, hyperchromatic, and pleomorphic nuclei. Mitotic figures are frequent and sometimes atypical. Single-cell necrosis and desquamation of necrotic cells in the lumen of the appendix may be present [10,57]. In high-grade appendiceal mucinous neoplasms, mutations in *SMAD4* (Mothers Against Decapentaplegic Homolog 4), *TP53*, and *APC* genes are more common than in low-grade appendiceal mucinous neoplasms. However, mutations in *FAT4*, *SMAD2*, *AKT1*, *MET*, *JAK3*, *PIK3CA*, *STK11*, *RB1*, and *RNF43* genes have also been identified, as well as the coexistence of mutations in *KRAS*, *NRAS*, and *RNF43* genes, *KRAS* and *TP53 IHC* genes, generally predominating C>T transitions. The lower frequency of mutations in the *GNAS* gene indicates the very low probability that they originate from low-grade appendiceal mucinous neoplasms [10,35,53].

### 5.3. Appendiceal Adenocarcinomas NOS and Undifferentiated Carcinomas NOS

**Figure 4:**
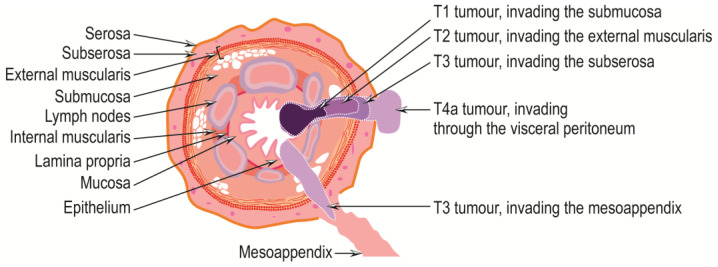
Definition of primary tumor (T1–T4a) in appendix adenocarcinomas.

**Figure 5:**
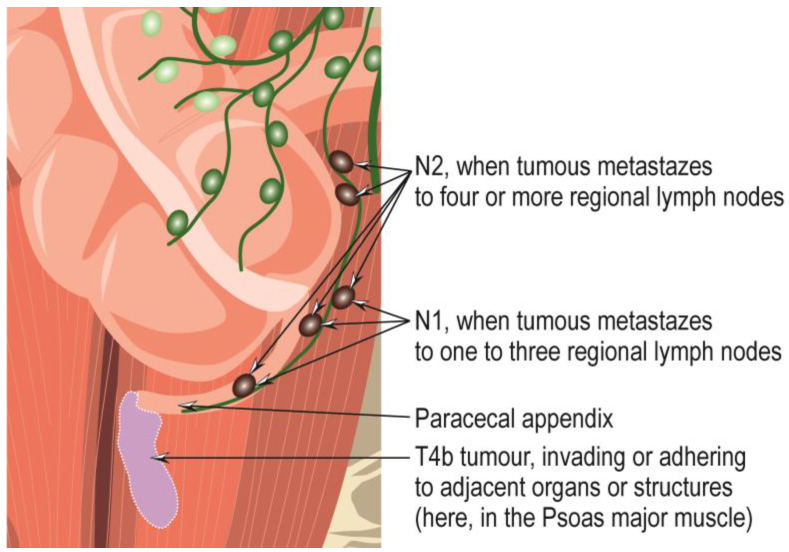
Definition of primary tumor (T4b) and regional lymph node (N1–N2), in appendix adenocarcinomas.

Appendiceal adenocarcinomas are malignant invasive glandular neoplasms that rarely occur, with a frequency of approximately 0.08% of all surgically excised appendicitis and 0.2–0.5% of all gastrointestinal neoplasms [58,59,60,61,62]. They are of unknown etiology, with previous appendiceal lesions as likely precursors [63]. They mainly affect patients in the fifth to seventh decades of life, with mucinous and signet cell forms being somewhat more common in women and non-mucinous forms in men [10]. They can occur anywhere within the appendix and can be symptomatically mistaken for acute appendicitis, so they are challenging to diagnose in the early stages, especially preoperatively [10,62]. However, the persistence of symptoms of acute appendicitis for a long time may indicate the presence of appendiceal adenocarcinoma [62]. Later, affected individuals may experience abdominal pain or present with a palpable mass, intestinal obstruction, intestinal bleeding, or other symptoms due to the development of metastases [10], which appear, in the first stage, in the ileocolonic nodal basin, as well as in the infraduodenal and para-aortic areas via the lymphatic pathway [62]. The tumor may be polyp-like, ulcerative, or infiltrative, with obstruction of the lumen and dilatation or perforation of the appendix [10]. According to ICD-0 coding, adenocarcinoma of the appendix NOS is designated 8140/3, and according to ICD-11 coding, adenocarcinoma of the appendix is designated 2B81.0, and mucinous adenocarcinoma of the appendix is designated 2B81.1. Adenocarcinomas of the appendix are classified into the following subtypes: the signet-ring cell adenocarcinoma of the appendix, coded 8490/3; mucinous adenocarcinoma, coded 8480/3; and undifferentiated carcinoma NOS, coded 8020/3. The criteria for the diagnosis and staging of appendiceal adenocarcinomas have been summarized, revised, and updated in the 8th edition of the *American Joint Committee on Cancer Staging Manual* (published in 2017) [64], and the 5th edition of the *WHO Classification of Tumours of the Digestive System* (published in 2019) [10] are reproduced in Table 1, Table 2, Table 3, Table 4 and Table 5 and shown in Figure 4 and Figure 5. Overall, the five-year survival rate for people with appendiceal carcinoma is 19–55% [10,65,66], and are higher for patients with mucinous tumors without carcinomatosis compared to those with non-mucinous carcinomas [67].

In most cases, non-mucinous adenocarcinoma of the appendix NOS produces acute appendicitis-like symptoms. Histologically, it presents with irregularly shaped or serrated glands infiltrating the appendix wall. In most cases, the mucinous glands are lined with columnar cells and excrete a reduced amount of mucin. This morphology brings this type of neoplasia closer to colorectal adenocarcinoma (sometimes classified as non-mucinous adenocarcinoma of the colorectal appendix type), from which it is differentiated by more aggressive growth and more frequent metastasis to lymph nodes [10,68]. At a distance, non-mucinous adenocarcinoma of the appendix NOS metastasizes to the liver and lung, and most cases are poorly or moderately differentiated [69]. Non-mucinous adenocarcinomas of the appendix are classified using a two-tier system, as in colorectal adenocarcinomas [10], and may frequently exhibit microsatellite instability [10,70].

Mucinous adenocarcinomas of the appendix are obstructive dilatations and occur by the intraluminal accumulation of mucoid material [71]. Histologically, they resemble low-grade appendiceal mucinous neoplasms, from which they are differentiated by expansile mucin blisters, which may occupy more than half of the tumor volume, in which bands, glands, or clusters of mucinous epithelial cells and atypical neoplastic cells float. Because mucinous adenocarcinomas of the appendix frequently metastasize to the peritoneum, most patients develop peritoneal pseudomyxoma [69], which can be fatal if left untreated. Mucinous tumors of the appendix are classified according to a three-grade system: grade 1—low-grade appendiceal mucinous neoplasms; grade 2—mucinous adenocarcinomas; and grade 3—signet-ring cell adenocarcinoma, the latter presenting large clusters of signet-ring cells floating in the mucinous pools and occupying more than half of their volume [10]. Genetically, mucinous adenocarcinomas of the appendix have mutations in exon 2 of the *KRAS* gene, sometimes in association with mutations in the *RNF43* gene or with co-mutations in the *GNAS* gene, mutations in the *GNAS*, *NRAS*, *PIK3CA*, and *AKT1* genes, or mutations in the *BRAF* gene in association with mutations in the *p53 IHC* and *RNF43* genes, sometimes bearing no mutations at all [10,35,53,54,72,73]. Co-mutations in other genes, such as the tumor suppressor gene *SMAD4*, are common in high-grade mucinous appendiceal adenocarcinomas, probably favoring the progression of indolent low-grade neoplasms into aggressive high-grade adenocarcinoma [53].

Some mucinous adenocarcinomas of the appendix can exhibit microsatellite instability [74]. In some instances, the appendix attaches itself to the surrounding organs or often fistulize internally inside the body, into the bladder, ileum, umbilicus, aorta, sigmoid colon, cecum, etc. [75], and to a small extent, externally, through the skin. In a previous paper, we presented the case of a 63 years old woman with a low-grade appendiceal mucinous neoplasm attached to the psoas major muscle, with a clinical picture of a psoas primary abscess (pseudomyxoma retroperitonei), which later fistulized into the abdominal wall and skin, with multiple recurrences.

Patients with tumors with lower histological grades have better 5-year survival rates than those with more advanced grades. Thus, the highest 5-year survival rate of 30–60% is for patients with mucinous adenocarcinomas, which drops significantly to 20–30% for those with signet-ring cell adenocarcinoma [10,76].

Signet-ring cell adenocarcinoma presents large clusters of signet-ring cells floating in the mucinous pools, occupying more than half of their volume [10]. Because of their advanced stage (grade 3), they have a poor prognosis. The association with genetic causes is not well known, with only a few cases reported in which mutations occurred in the *CDH1* (Cadherin 1) gene when appendiceal signet-ring cell adenocarcinoma was associated with gastric signet-ring cell adenocarcinoma [77], and also in the *KRAS* [72,78] and *GNAS* genes [10,72].

Undifferentiated carcinomas of the appendix are rare, scarcely mentioned in the literature, and have a histological appearance similar to other undifferentiated carcinomas of the colon and rectum [10].

### 5.4. Appendiceal Goblet Cell Adenocarcinoma

First described in 1974 [79], appendiceal goblet cell adenocarcinoma is a unique amphicrine tumor originating in pluripotent cells with neuroendocrine and mucinous differentiation; it is characterized by intense proliferation due to cell cycle disruption through upregulation of cyclin D1 and p21 and downregulation of p16 [80], and occurs almost exclusively in the distal part of the appendix. Histologically, goblet cells predominate, along with a few neuroendocrine cells and occasionally paneth-like cells with granular eosinophilic cytoplasm, arranged in discrete, tubular, intestinal crypt-like nests that arise deep in the lamina propria and develop concentrically in the wall of the appendix [10,81,82]. Low-grade tumors appear as tubules of goblet-like mucinous cells and variable numbers of endocrine and paneth-like cells. Some tumor cell clusters are small groups of cohesive goblet-like cells that are devoid of light. Mild disorganization and tubular fusions, mild nuclear atypia, and rare mitoses may also be seen [10], since extracellular mucin, sometimes abundant, is always present [83]. High-grade tumors include tumor infiltrates composed of single cells, complex anastomoses of tubules, cribriform masses, sheets of tumor cells, and large clusters composed of goblet or signet-ring cells. A desmoplastic stromal response, numerous atypical mitoses, and necrosis may also be present, with tumors sometimes having a typical adenocarcinoma appearance, with irregular glands bordered by malignant-looking columnar cells. Perineural invasions are present frequently, irrespective of tumor grade, whereas lymphovascular invasions are common only in high-grade tumors. Appendiceal goblet cell adenocarcinoma equally affects adults of both sexes aged 30 to 85 years, peaking in the sixth decade of life and, depending on the grade of the tumor, it has biological behavior ranging from indolent to more aggressive than that of simple appendiceal adenocarcinomas [10,82,84,85]. Affected patients present with non-specific or, at most, appendicitis-like symptoms. The tumor may be discovered incidentally during abdominal imaging explorations as an abdominal mass, especially in women with ovarian metastases or appendectomies performed for other reasons. In these cases, the appendix may range from standard to wall thickening, with high-grade tumors with an infiltrative and indurated appearance. According to ICD-0 coding, appendiceal goblet cell adenocarcinoma is designated as goblet cell adenocarcinoma, 8243/3, and according to ICD-11 coding, as malignant neoplasms of the appendix and goblet cell adenocarcinoma, 2B81 and XH4262.

Appendicular goblet cell adenocarcinoma has no subtypes and is staged similarly to appendiceal adenocarcinoma NOS through a three-tiered system that considers the proportion of tubular or clustered growth expansion as a low-grade feature and loss of growth as a high-grade feature. Thus, grade 1 is marked by more than 75% tubular or clustered growth, grade 2 by 50–75% tubular or clustered growth, and grade 3 by less than 50% tubular or clustered growth [10,82,83]. The TNM staging is similar to other appendiceal tumors. The 5-year overall survival is around 75%, and the most important factor for prognosis is tumor TNM stage: for stage I, it is 100%; for stage II, 76%; for stage III, 22%; and for stage IV, 14% [86,87,88]. Typically, patients with grade 1 tumors have an extended life expectancy of 84 to 204 months, those with grade 2 tumors 60 to 86 months, and patients with disseminated and aggressive grade 3 tumors survive between 29 and 45 months [81,82,89,90]. In the latter, cytoreductive treatment combined with HIPEC has no effect in terms of increasing life expectancy [10,89]. Generally, patients with low-grade appendiceal goblet cell adenocarcinoma are included in stage I or II, although some may develop metastases in the peritoneum, which can be managed with cytoreductive surgery and heated intraperitoneal chemotherapy, in the omentum, in the abdominal wall, and in the ovaries, whereas 50 to 70% of patients with high-grade tumors are included in stage IV [81,82,87,89,91].

Genetically, goblet cell adenocarcinomas of the appendix are characterized by mutations in the *TP53* gene, which is present mainly in high-grade goblet cell adenocarcinomas [10,89,92], in *SMADA* and *KRAS* genes [93], although the latter appears to be less or not at all important in the development of these tumors, unlike *P53*, which seems to play an essential role in their development [94], in *USP9X* (Ubiquitin Specific Peptidase 9 X-Linked), *NOTCH1* (Notch Receptor 1), *CTNNA1* (Catenin Alpha 1), *CTNNB1* (Catenin Beta 1) and *TRRAP* (Transformation/Transcription Domain Associated Protein) genes, which are part of the WNT signaling pathway [10,95], involved in the ability of cells to renew and differentiate, also playing a very important role in the development of liquid and solid tumors [96], and, less commonly, in genes involved in chromatin remodeling, including *ARID1A* (AT-Rich Interaction Domain 1A), *ARID2* (AT-Rich Interaction Domain 2), *KDM6A* (Lysine Demethylase 6A) and *KMT2D* (Lysine Methyltransferase 2D) [92,93,97]. Similarly to mutations in the *KRAS* and *SMAD4* genes, mutations in the *APC* gene, all typical for colorectal cancers, are rare in goblet cell adenocarcinomas of the appendix [92,97].

### 5.5. Appendiceal Neuroendocrine Neoplasms

Along with similar neoplasms of the colon and rectum, neuroendocrine neoplasms (NENs) of the appendix show morphological and immunophenotypic features of neuroendocrine differentiation and histopathological examination reveals the existence of neuroendocrine cell populations in a proportion of more than 30%. According to the WHO classification, neuroendocrine neoplasms (NENs) of the appendix (A-NEN or a-NEN) include well-differentiated neuroendocrine tumors (NETs), poorly differentiated neuroendocrine carcinomas (NECs) and mixed neuroendocrine neoplasms (MiNENs), which, in addition to the neuroendocrine component, also include a non-neuroendocrine component, usually corresponding to a mucinous or non-mucinous adenocarcinoma. Both can be recognized as distinct and discrete components, each comprising at least one-third of the lesion. The behavior of neuroendocrine neoplasms (NENs) of the appendix is relatively indolent in the case of well-differentiated neuroendocrine tumors, which become aggressive in a small number of cases, or aggressive in the case of poorly differentiated neuroendocrine carcinomas and mixed neuroendocrine neoplasms [10,98,99]. Neuroendocrine tumors of the appendix are relatively common, have an incidence of 0.15–0.6 cases per 100,000 persons/year, and affect people of both sexes, with a maximum incidence in those under 40 years of age and a slight female predominance [100].

The criteria for diagnosing and staging appendiceal neuroendocrine neoplasms have been summarized, revised, and updated in the 8th edition of the *American Joint Committee on Cancer Staging Manual* (published in 2017) [64]. In addition, the 5th edition of the *WHO Classification of Tumours of the Digestive System* (published in 2019) [10] is reproduced in Table 6, Table 7, Table 8 and Table 9.

Well-differentiated neuroendocrine tumors in the appendix are classified according to the overall incidence of neuroendocrine tumors in the appendix [99,101]. However, they may also occur frequently in children, in whom long-term outcomes are excellent, with appendectomy being sufficient and curative, without influence on life expectancy, and without causing death, regardless of size, invasiveness, tumor spread, and subsequent treatment, and no need to institute chemotherapeutic treatment [10,102,103,104,105]. So far, it is unclear whether right hemicolectomy can offer any advantage [106], but it seems to be indicated for tumors larger than 1 cm [107]. Well-differentiated neuroendocrine tumors of the appendix consist of uniform populations of cells with spherical nuclei and finely punctate chromatin. The cells are organized into trabecular, acuminate, filiform, or nest-like structures, and generally arise at the tip of the appendix. Less often, they develop in the base or body of the appendix when they obstruct the appendix lumen and appendicitis. Moreover, well-differentiated neuroendocrine tumors of the appendix have no specific symptoms, apart from those common to acute appendicitis (acute, subacute, or chronic abdominal pain), with which they are often confused, and they are discovered accidentally only after the pathological examination of the specimens of appendix removed for inflammation.

Rarely, well-differentiated neuroendocrine tumors of the appendix may be associated with carcinoid syndrome, indicating disease progression and metastatic spread. The subsequent outcome is favorable, with a survival rate of more than 90% of patients ten years after diagnosis and a risk of metastasis o regional lymph nodes of less than 10%. Lymph node metastasis does not necessarily lead to reduced life expectancy [10,102,104]. Liver or other organ metastases are rare, as indicated by the primary tumor’s size, and lead to a decreased 5-year survival rate of 34.8% [108]. Thus, the metastatic potential of tumors less than 1 cm in size is 0–11%, those of 1–2 cm in size increase to 18–44%, and those greater than 2 cm is 30–86% [10,109,110,111]. Based on the size, well-differentiated neuroendocrine tumors of the appendix are classified in the TNM system as T1 when they are less than 2 cm in size; T2 when they are 2–4 cm in size; T3 when they are more than 3 cm in size or invade the subserosa or mesoappendix, regardless of tumor size; and T4 when they perforate the serosa or directly invade adjacent organelles and structures [98]. Because most well-differentiated neuroendocrine tumors are small in size and invade the subserosa and mesoappendix, they are classified as pT3 [10] and, based on the mitotic index and the proliferative index Ki-67, are categorized via the three-tiered system into three grades: G1, with serotonin-producing enterochromaffin-cells; G2 and G3, with L-cells, which secrete chromogranin-B; and other hormone-producing cells [98]. Grade 1 tumors are the most common, which occur in the deep muscle layer and subserosa, and comprise polygonal cells arranged in large nests, often with peripheral palisading and glandular formations, with frequent fibrotic stromal responses, rare mitosis when present, and occasional necrosis. In the deep muscle layer, the nests are replaced by short ribbons of tumor cells. In about 33% of the cases, grade 1 tumors shed infiltrates into the mesoappendix. More rarely, tumors with few L-cells classified as grades 2 and 3 have trabecular or glandular growth and produce glucagon-like peptide-1 (GLP-1) and other proglucagon-derived peptides. In a few cases, well-differentiated neuroendocrine tubular tumors may develop [10,98,112]. Genetically, well-differentiated neuroendocrine tumors of the appendix seem to be associated with mutations in *TP53*, *PTEN*, and *EGFR* genes [94,98] but not with *APC*, *BRAF*, and *PIK3CA* gene mutations [97]. The existence of mutations in the *SMAD4* gene is still unclear.

On the other hand, poorly differentiated neuroendocrine carcinomas and mixed neuroendocrine neoplasms are sporadic. Poorly differentiated neuroendocrine carcinomas have small cells, giant cells, or a mixture of the two, are poorly differentiated, organized in trabeculae or sheets, and have a high mitotic rate and Ki-67 proliferation index. Poorly differentiated neuroendocrine carcinomas and mixed neuroendocrine neoplasms have morphology identical to colon carcinomas, may originate from precursor mucosa lesions, are more aggressive, and have behavior and progression common to other appendiceal and colon carcinomas. Compared to well-differentiated neuroendocrine tumors, mixed neuroendocrine neoplasms are associated with reserved and unfavorable prognoses but are better than adenocarcinomas of the appendix. However, in the advanced stages, their evolutions become similar. As a result, the 5-year survival rate is comparable to that of their counterparts in other parts of the gastrointestinal tract. Although rare, poorly differentiated neuroendocrine carcinomas and mixed neuroendocrine neoplasms have not yet been characterized in terms of mutations [10,98,100,113,114,115].

### 5.6. Therapeutic Approaches for Appendiceal Cancers

Usually, acute appendicitis is treated with the surgical resection of the inflamed appendix [116] using a classical open or laparoscopic approach or with several alternative therapies, including antibiotic administration [117,118] and endoscopic retrograde appendicitis therapy (ERAT) [2]. Table 10 summarizes their effectiveness, benefits, and limitations.

The first report on the surgical treatment of acute appendicitis dates from 1736, when Claudius Amyand operated on an 11-year-old boy with a scrotal hernia (which is called Amyand hernia, with four subtypes; see further), who also had the appendix inflamed and perforated by a needle encrusted with a stone towards the head, located in the hernial sac. After a month of bed rest and following a strict regimen, the boy was considered recovered [116]. The next case of appendectomy for suspected acute appendicitis was reported by Robert Lawson Tait in 1880. Appendectomy should be performed as soon as possible after the onset of symptoms to avoid complications, the most common of which are gangrene and perforation of the appendix wall. These occur more frequently in children and the elderly. Perforation of the appendix leads to spillage of its contents into the abdominal cavity and infection of the peritoneum with microorganisms (peritonitis), which is more challenging to treat and can be life-threatening. In addition, when lumen obstruction is caused by hypertrophy of the lymph nodes, resolution may occur spontaneously [11].

In 1910, spontaneous resolution of acute appendicitis identified in an uneviscerated mummy belonging to a Nubian woman from the Byzantine era was reported [128]. In 1930, Hamilton Bailey proposed a non-operative treatment algorithm for acute appendicitis [133]. The first study on the use of antibiotics for the conservative treatment of appendicitis was conducted by Coldrey [134]. It included 471 patients treated only intravenously with antibiotics, 48 requiring an appendectomy, 9 requiring abscess drainage, and 1 patient dying. This indicated that antibiotics have proven to be an alternative treatment for acute appendicitis [122]. In 2020, the results of a pragmatic, nonblinded, noninferiority, randomized trial conducted in 25 centers in the United States comparing the effectiveness of appendectomy and antibiotic therapy administered for ten days were published [127]. Of the 1552 patients with acute appendicitis, 776 underwent appendectomy (of which, 96% underwent laparoscopic surgery) and 776 received antibiotic therapy. After 30 days, the effectiveness of antibiotic use was comparable to that of appendectomy. However, 90 days after the start of the study, 29% of those treated with antibiotics required an appendectomy, including 41% of those with appendicoliths and 25% without appendicoliths. Regarding adverse events, these were 4% in the antibiotic-treated group and 3% in the appendectomy group. The conclusion of the study reinforced what had been stated earlier [122,126,134], indicating that the use of antibiotics in the treatment of uncomplicated acute appendicitis (appendicitis without perforation, appendiceal abscess, or mass formation) may be an alternative to appendectomy [122,123,126,127,134]. At five years from the onset of symptoms, recurrence of acute appendicitis was reported in 39.1% of the patients treated with antibiotics alone [2,117,118].

Endoscopic retrograde appendicitis therapy (ERAT) assumes an endoscopic intervention for draining pus, extracting fecoliths, and stenting when necessary. Employing this strategy, up to 95% of patients were registered with no recurrence [2]. Other advantages of ERAT compared to open or laparoscopic appendectomy include preserving the appendix, reduced trauma, faster recovery times, and lower costs [129,130]. Moreover, ERAT facilitates the precise diagnosis of acute appendicitis and is a potential diagnostic tool for patients with atypical clinical manifestations [130,131]. Nonetheless, extensive clinical studies should be performed before the general adoption of these non-surgical treatments in terms of efficacy and long-term safety; for instance, one study has linked the use of antibiotics and drainage procedures with an increased incidence of bowel cancer [132].

The eight types of appendiceal cancers are included in five main histopathological subtypes with two origins: mucinous neoplasms, goblet cell adenocarcinomas (GCAs), nonmucinous appendiceal adenocarcinomas and signet ring cell adenocarcinomas, with epithelial origin, and neuroendocrine neoplasms (NENs), which have a non-epithelial origin [34]. Because appendiceal cancers are rare pathological entities with features that resemble the corresponding colorectal cancers, data on therapeutic approaches strictly for appendiceal cancers are limited, with therapies mainly similar to those applicable to colorectal tumors. The therapeutic strategies for each histological type of appendiceal cancer are summarized in Table 11. Thus, for tumors of epithelial origin, which frequently metastasize into the peritoneal cavity and rarely outside the peritoneal cavity, the most commonly used approach is the combination of cytoreductive surgery (CRS) and HIPEC. This method’s advantage is removing any visible tumors from the intraperitoneal space by cytoreductive surgery and inactivating microscopic tumor cell deposits by hyperthermic intraperitoneal chemotherapy [135]. The application of the warm chemotherapeutic agent allows tissue penetration up to 0.5–5 mm in depth, killing the residual tumor cells within this layer [136]. However, it decreases peritoneal carcinomatosis, to which appendiceal and colorectal cancers of epithelial origin progress; it cannot prevent recurrences or guarantee long-term survival [135].

For localized mucinous neoplasms that do not penetrate the appendix wall and are diagnosed intraoperatively or postoperatively, the surgical procedure aims to remove the neoplastic appendix intact without allowing the spread of malignant cells [34]. In addition to appendectomy, right hemicolectomy is recommended in cases of grades 2 and 3 mucinous neoplasms with lymph node involvement. These may be sufficient for a complete cure or long-term disease control. Intraoperatively, it is necessary to search for, identify, and remove any mucinous collections in the pelvis, omentum, lateral paracolic recesses, and diaphragmatic abdominal surface [78,137]. When these have acellular mucin, the risk of pseudomyxoma peritonei is reduced, but it is increased in deposits with cellular mucin [137]. To treat pseudomyxoma peritonei, a combination of cytoreductive surgery and hyperthermic intraperitoneal chemotherapy is recommended. Complete cytoreduction may require six separate operations, in which the following are removed: (i) greater omentum and spleen; (ii, iii) upper peritoneum (left and right); (iv) lesser omentum, gallbladder, and omental bursa; (v) pelvic peritoneum and sigmoid colon; (vi) distal third of the stomach (the gastric or pyloric antrum). These therapies are standardized and accepted worldwide [138]. Systemic chemotherapy does not bring substantial benefit for grade 1 mucinous neoplasms/pseudomyxoma of the peritoneum. However, fluorouracil-based chemotherapy is recommended for grades 2 and 3 of mucinous neoplasms/pseudomyxoma of the peritoneum, as in colorectal cancers. Perioperative systemic chemotherapy may reduce the intra-abdominal spread of the tumor and improve the quality of life [139,140,141,142]. For mucinous adenocarcinomas, which are invasive mucinous neoplasms, the standard surgical treatment includes right hemicolectomy, although its effectiveness is not entirely accepted [143] due to the high recurrence rate. Better results, in terms of disease remission and prolongation of the disease-free period, are achieved by chemotherapy [144] and mainly by the combination of repeated cytoreductive surgery and hyperthermic intraperitoneal chemotherapy, even in the presence of a large initial volume [145].

The treatment of goblet cell adenocarcinomas identified after appendectomy and without visible carcinomatosis includes right hemicolectomy [146]. When carcinomatosis develops, the combination of cytoreductive surgery and hyperthermic intraperitoneal chemotherapy prolongs the median overall survival from 18 to 37 months and 4-year survival rates up to 24% [147]. In stage III and IV goblet cell adenocarcinoma cases, adjuvant chemotherapy with 5-fluorouracil-based products improves survival duration [148] (Table 11).

Nonmucinous appendiceal adenocarcinomas are similar to colonic adenocarcinomas and are referred to as colonic-type appendiceal adenocarcinomas. The treatment strategy for nonmucinous appendiceal adenocarcinomas is similar to that for signet ring adenocarcinomas and goblet cell adenocarcinomas, including right hemicolectomy [149], followed by standard systemic chemotherapy. When cytoreductive surgery/hyperthermic intraperitoneal chemotherapy is administered, the survival of patients with appendiceal adenocarcinoma is improved, and peritoneal metastasis is lower compared to systemic chemotherapy alone [150,151,152]. Systemic chemotherapy is unnecessary for metastasis-free disease without lymph node involvement and is limited to the appendix only. Localized, completely resected adenocarcinomas with lymph node involvement respond well to adjuvant fluoropyrimidine/oxaliplatin chemotherapy. Because metastatic adenocarcinomas of the appendix do not benefit from standardized chemotherapeutic treatment, they are treated with treatment regimens common to those for colon adenocarcinomas, namely combinations of fluorouracil, platinum, and irinotecan [153,154].

Neuroendocrine neoplasms frequently metastasize to lymph nodes in a manner depending on the tumor size. Small stage I and II neuroendocrine neoplasms with minimal lymph node involvement (N0 or N1) have a good prognosis, with 5- and 10-year survival rates of 100%. Their treatment, when less than 2 cm in size, is an appendectomy, irrespective of the depth of mesoappendix invasion (>3 mm), the presence of positive or unclear margins, lymphovascular invasion, and proliferation rate. In these cases, right hemicolectomy does not offer substantial benefit. For localized tumors larger than 2 cm, appendectomy and right hemicolectomy are indicated. Tumors with endocrine hypersecretion are at risk of metastasis [155]. In evaluating and determining the therapeutic management of patients with metastatic appendiceal neuroendocrine neoplasms, the site of metastasis (hepatic or other sites), tumor load, and somatostatin hormone secretion status are considered [156]. The treatment of metastatic tumors is complex and includes cytoreductive surgery for resectable tumors, including liver metastatic tumors, relieving symptoms, and improving long-term survival [155,157]. For unresectable liver metastases, therapeutic strategies include bland hepatic artery embolization, intra-arterial chemoembolization with cisplatin or doxorubicin, and 90Y-radioembolization [158]. Other therapeutic strategies include using somatostatin analogs, such as octreotide (in the PROMID phase III study) and lanreotide (in the CLARINET phase III study). These drugs increase the time to metastasis [159]. Targeted therapies, such as everolimus (inhibitor of the mTOR signaling pathway), sunitinib (inhibitor of VEGF receptors 1–3), and surufatinib (inhibitor of angiogenesis, by inhibiting VEGFRs and FGFRs), reduce the ability of tumor cells to multiply [155]. The most widely used chemotherapeutic agents in the treatment of differentiated neo-endocrine tumors are FOLFOX (folinic acid, fluorouracil, and oxaliplatin) and XELOX/CAPOX (capecitabine and oxaliplatin) combinations, which are based on oxaliplatin, with anti-tumor activity in advanced cases of gastrointestinal neo-endocrine tumors [160,161], and CAPTEM (capecitabine and temozolomide) combination [162], with minor benefits. Some studies have tried combining FOLFOX or XELOX/CAPOX with bevacizumab [163,164], but the results were comparable to administering FOLFOX or XELOX/CAPOX alone [164].

**Table 11:** Therapeutic approaches for appendiceal cancers.

Tumor Type	Therapeutic Approaches
Mucinous neoplasms, grade 1	Appendectomy [34]
Mucinous neoplasms, grades 2, 3	Appendectomy + right hemicolectomy + perioperative systemic chemotherapy [137,141,142]
Pseudomyxoma peritonei	Cytoreductive surgery + hyperthermic intraperitoneal chemotherapy [138]
Pseudomyxoma peritonei, grades 2, 3	Cytoreductive surgery + hyperthermic intraperitoneal chemotherapy [138] + perioperative systemic chemotherapy [139,140,142]
Mucinous adenocarcinoma	Right hemicolectomy [143] Chemotherapy [144] Cytoreductive surgery + hyperthermic intraperitoneal chemotherapy [145]
Goblet cell adenocarcinomas without carcinomatosis	Right hemicolectomy [146]
Goblet cell adenocarcinomas with carcinomatosis	Cytoreductive surgery + hyperthermic intraperitoneal chemotherapy [147]
Goblet cell adenocarcinomas, stages III, IV	Cytoreductive surgery + adjuvant chemotherapy [148]
Nonmucinous appendiceal adenocarcinomas/signet ring adenocarcinomas without metastases	Right hemicolectomy [149]
Nonmucinous appendiceal adenocarcinomas/signet ring adenocarcinomas with metastases	Right hemicolectomy [149] + standard systemic chemotherapy (fluorouracil, platinum, and irinotecan [153]) + cytoreductive surgery/hyperthermic intraperitoneal chemotherapy [150,151,152]
Localized nonmucinous appendiceal adenocarcinomas/signet ring adenocarcinomas without node involvement	Right hemicolectomy [149] + adjuvant chemotherapy (fluoropyrimidine/oxaliplatin) [153]
Neuroendocrine neoplasms < 2 cm	Appendectomy [155]
Neuroendocrine neoplasms > 2 cm	Right hemicolectomy [155]
Resecable metastatic neuroendocrine neoplasms	Cytoreductive surgery [155,157]
Non-resectable metastatic neuroendocrine neoplasms	Bland hepatic artery embolization + intra-arterial chemoembolization with cisplatin or doxorubicin + and ^90^Y-radioembolization [158] Somatostatin analogue therapies: octreotide (PROMID phase III study)/lanreotide (în CLARINET phase III study) [159] Everolimus (mTOR inhibitor)/Sunitinib (VEGFRs 1–3 inhibitors), and surufatinib (angiogenesis inhibitor) [155] FOLFOX (folinic acid, fluorouracil and oxaliplatin)/XELOX/CAPOX (capecitabine and oxaliplatin) [160,161] CAPTEM (capecitabin and temozolomide) [162] FOLFOX or XELOX/CAPOX + bevacizumab [163,164]

## 6. Other Diagnoses Associated with the Vermiform Appendix

Several rare conditions linked to the vermiform appendix have been reported in the literature without fitting into any of the three main categories presented above. A retrospective cross-sectional study revealed that some appendectomy specimens were infested with *Enterobius vermicularis*, i.e., pinworm infection [165]. Other parasites reportedly found in the vermiform appendix include *Taenia* spp. (tapeworm), *Hymenolepis nana* (dwarf tapeworm), *Ascaris lumbricoides* (roundworm), *Trichuris trichiura* (whipworm), *Schistosoma haematobium*, *Schistosoma mansoni*, *Giardia lamblia*, and the protozoan *Entamoeba histolytica* [165,166]. Such parasite infections may further cause appendicular colic [167] or appendicitis [167,168,169].

The vermiform appendix may also rarely be prone to fungal infections, especially in immunocompromised patients. Fungal microorganisms may colonize the appendiceal mucosa or the periappendiceal vessels, eventually leading to fungal appendicitis, which is no different from acute appendicitis concerning clinical symptoms. Case reports include mucormycosis, histoplasmosis, aspergillosis, and candidiasis as the microbial causes of fungal appendicitis [170,171,172].

Another rare condition of the vermiform appendix is endometriosis [165]. Appendiceal endometriosis may be asymptomatic or may cause similar manifestations of acute and chronic appendicitis, being also associated with cyclic and chronic right lower quadrant pain, melena, lower intestinal hemorrhage, cecal intussusception, and intestinal perforation [173,174].

Appendiceal intussusception is another infrequent pathology of the vermiform appendix. It supposes invagination, to various degrees, of the appendix into the cecum and may escalate to ileocecal intussusception, masking the underlying problem. Symptoms may mimic acute appendicitis, but patients may also experience remitting abdominal pain, vomiting, diarrhea, and rectal bleeding. In addition to appendectomy, the management of appendiceal intussusception also involves the removal of the rim of the cecum at the base of the appendix to prevent recurrence [175,176,177].

## 7. Discussion

Intraoperative procedures for peritoneal surface malignancy have significantly improved via collaborative efforts among palliative centers worldwide. This progress has been facilitated by sharing knowledge, experiences, and techniques, leading to refining and standardizing surgical procedures. The process involves peritonectomy (removal of the peritoneum affected by cancer) and organ resection, which have been thoroughly described and standardized in the comprehensive resource *Cytoreductive Surgery & Perioperative Chemotherapy for Peritoneal Surface Malignancy: Textbook and Video Atlas* [178]. This textbook provides detailed illustrations and guidance to help maintain consistency and uniformity in these complex surgical procedures.

The publication of various palliative care textbooks and the continuous refinement and updating of expert consensuses have significantly contributed to the standardization of Cytoreductive Surgery (CRS). CRS is a crucial component of the comprehensive treatment approach for peritoneal surface malignancy, aiming to remove visible tumor nodules and achieve optimal cytoreduction.

However, despite these significant advances, some controversies still exist concerning the application of HIPEC. The controversy likely arises from variations in patient selection, chemotherapy agents used, optimal dosages, and the exact duration of HIPEC treatment. As stated in Table 11, in appendiceal neoplasms, HIPEC is indicated in pseudomyxoma of the peritoneum, mucinous adenocarcinoma, goblet cell adenocarcinomas with carcinomatosis, and nonmucinous appendiceal adenocarcinomas/signet ring adenocarcinomas with metastases. However, some opinions question the benefits it offers. HIPEC consists of the intraperitoneal introduction of a warm (42 °C) solution containing a cytotoxic agent, and, indeed, it targets cell collections that cytoreduction has missed because of their microscopic size or inaccessibility. Due to heating, the solution can deliver the cytotoxic agent up to 5 mm deep into tissues. For 5-fluorouracil, this penetration is only 0.5 mm; for cisplatin, 1–5 mm; for carboplatin, 0.5 mm; for oxaliplatin, 1–2 mm; and for mitomycin C, 2–5 mm, with the last two compounds being preferred. For other agents, the values are intermediate [136]. Controversies related to this therapy come from the different results obtained by the studies. Studies with positive results come in support of the use of HIPEC [145,147,150,151,152,179]. On the other hand, several recent phase III randomized trials show that HIPEC does not benefit disease progression-free survival or overall survival. The Dutch COLOPEC trial, conducted in patients with resected T4 or perforated colon cancer, examined the differences in peritoneal metastasis-free survival at 18 months with or without adjuvant HIPEC with oxaliplatin for 30 min, with no notable differences [180]. Two French studies followed this. PROPHYLOCHIP-PRODIGE 15 tested the efficacy of cytoreductive surgery and HIPEC (predominantly oxaliplatin) administered after six months of adjuvant systemic chemotherapy to colorectal cancer cases with synchronous and localized peritoneal metastases, removed with the primary tumor, with perforated primary tumor or with resected ovarian metastases free of recurrence, which failed to achieve superior results to cytoreductive surgery and adjuvant systemic chemotherapy [181]. This study was followed by the PRODIGE 7 trial, which failed to achieve increased overall survival in patients with colorectal peritoneal metastases and treated with cytoreductive surgery and HIPEC compared to those treated with cytoreductive surgery alone [182].

Although the results appear to be conflicting, it is clear that for some patients, even with extensive carcinomatosis and high-grade tumors, HIPEC may bring benefits. In this sense, a French study has drawn a linear relationship between Peritoneal Carcinomatosis Index and survival [183], showing that combining cytoreductive surgery with HIPEC is indicated for Peritoneal Carcinomatosis Index as less than 12 and contraindicated for values as greater than 17. Between these values, the success of the combined treatment depends on other parameters, the conclusion being that further investigations are needed to establish the effectiveness of cytoreductive surgery and HIPEC. Collaborative efforts among multidisciplinary teams will continue to play a crucial role in refining and standardizing HIPEC procedures, ultimately leading to better patient outcomes and quality of life for individuals with appendiceal malignancy.

## 8. Conclusions

Despite its small size (on average 9 cm in length and 6 mm in diameter), the vermiform appendix is an organ with several physiological roles and various pathologies, the most common of which is acute appendicitis. The other pathologies of the vermiform appendix, especially its neoplasia, are rare and often go unnoticed, being accidentally identified during appendectomies performed for other reasons. In the early stages, most appendiceal neoplasms are not detected, while in advanced stages, they may mimic the symptoms of acute appendicitis. In addition, due to massive mucus production, mucinous neoplasms, especially adenocarcinomas, may fistulize into adjacent structures, some identified after perforated organ pathology.

The general treatment of appendiceal pathologies, including neoplasms, is complete surgical excision of the appendix with or without right hemicolectomy. Life expectancy is somewhat longer for low-grade mucinous tumors and peaking for well-differentiated, small, metastasis-free neuroendocrine tumors of the appendix occurring in children. Since appendiceal tumors generally occur after the age of 40–50 years, in families with a history of it, their members should test at least for CEA (carcinoembryonic antigen) and CA-19-9 (carbohydrate antigen) markers annually, starting in the forties, for the early detection of any neoplastic transformation related to the gastrointestinal tract.

Moreover, supplementary research should be performed on alternative therapeutic strategies to clarify the long-term effectiveness of non-surgical approaches and establish new treatment possibilities. Future clinical studies should also report on atypical manifestations of vermiform appendix-related health conditions toward creating a comprehensive framework for clinicians to better understand, diagnose, and treat these pathologies and address each patient’s personalized needs.

## 9. Future Perspectives

Today, the embryology, anatomy, and much of the physiology of the appendix are well known, but some questions remain about appendix pathology, which further research may shed light on. One of these questions concerns the efficacy of appendectomy or endoscopic retrograde appendicitis therapy compared to the administration of antibiotics in cases of acute uncomplicated appendicitis. Although studies have been undertaken with encouraging results in favor of resolving acute appendicitis with antibiotics and endoscopic retrograde appendicitis therapy, more conclusive investigations are needed.

On the other hand, although rare, appendiceal tumors are sometimes disabling and have high mortality. Therapeutic strategies include appendectomy and chemotherapy in cases of localized tumors without metastases and lymph node involvement. In extensive tumors, especially mucinous ones, with appendiceal wall rupture and spreading muco-cellular contents into the pelvic cavity, treatment becomes complex, including right hemicolectomy and HIPEC, with no guarantee of success, especially in recurrent tumors. Framed together with cancers of the large intestine, appendiceal cancers are treated similarly, although there are some differences genetically and pathologically. Under these circumstances, it is necessary to further investigate the differences between the two groups of pathological entities and identify specific markers for appendiceal cancers.

It is also necessary to identify cases where the right hemicolectomy should be performed to obtain increased survival benefits and reduce the risk of recurrence and liver or peritoneal metastasis (pseudomyxoma peritonei). Finally, there is a need to clarify when HIPEC should be used and identify parameters indicating its success. In addition, it is necessary to investigate the effects of innovative and targeted therapies developed in recent decades on appendiceal cancers.

## Figures and Tables

**Table 1:** The criteria for the diagnosis and staging of appendiceal adenocarcinomas, according to the *AJCC Staging Manual* (2017) [64] and *WHO Classification of Tumours of the Digestive System* (2019) [10]: definition of the primary tumor (T).

T Category	T Criteria
TX	Primary tumor cannot be assessed
T0	No evidence of primary tumor
Tis	Carcinoma in situ (intramucosal carcinoma; invasion of the lamina propria or extension into but not through the muscularis mucosae)
Tis (LAMN)	Low-grade appendiceal mucinous neoplasm confined by the muscularis propria. Acellular mucin or mucinous epithelium may invade the muscularis propria. T1 and T2 are not applicable to LAMN. Acellular mucin or mucinous epithelium that extends into the subserosa or serosa should be classified as T3 or T4a, respectively.
T1	Tumor invades the submucosa (through the muscularis mucosa but not into the muscularis propria)
T2	Tumor invades the muscularis propria
T3	Tumor invades through the muscularis propria into the subserosa or the mesoappendix
T4	Tumor invades the visceral peritoneum, including the acellular mucin or mucinous epithelium involving the serosa of the appendix or mesoappendix, and/or directly invades adjacent organs or structures
T4a	Tumor invades through the visceral peritoneum, including the acellular mucin or mucinous epithelium involving the serosa of the appendix or serosa of the mesoappendix
T4b	Tumor directly invades or adheres to adjacent organs or structures

**Table 2:** The criteria for the diagnosis and staging of appendiceal adenocarcinomas, according to the *AJCC Staging Manual* (2017) [64] and *WHO Classification of Tumours of the Digestive System* (2019) [10]: definition of regional lymph node (N).

N Category	N Criteria
NX	Regional lymph nodes cannot be assessed
N0	No regional lymph node metastasis
N1	One to three regional lymph nodes are positive (tumor in lymph node measuring ≥ 0.2 mm), or any number of tumor deposits is present, and all identifiable lymph nodes are negative
N1a	One regional lymph node is positive
N1b	Two or three regional lymph nodes are positive
N1c	No regional lymph nodes are positive, but there are tumor deposits in the subserosa or mesentery
N2	Four or more regional lymph nodes are positive

**Table 3:** The criteria for the diagnosis and staging of appendiceal adenocarcinomas, according to the *AJCC Staging Manual* (2017) [64] and *WHO Classification of Tumours of the Digestive System* (2019) [10]: definition of distant metastasis (M).

M Category	M Criteria
M0	No distant metastasis
M1	Distant metastasis
M1a	Intraperitoneal acellular mucin, without identifiable tumor cells in the disseminated peritoneal mucinous deposits
M1b	Intraperitoneal metastasis only, including peritoneal mucinous deposits containing tumor cells
M1c	Metastasis to sites other than the peritoneum

**Table 4:** The criteria for the diagnosis and staging of appendiceal adenocarcinomas, according to the *AJCC Staging Manual* (2017) [64] and *WHO Classification of Tumours of the Digestive System* (2019) [10]: definition of histologic grade (G).

G	G Definition
GX	Grade cannot be assessed
G1	Well-differentiated
G2	Moderately differentiated
G3	Poorly differentiated

**Table 5:** The criteria for the diagnosis and staging of appendiceal adenocarcinomas, according to the *AJCC Staging Manual* (2017) [64] and *WHO Classification of Tumours of the Digestive System* (2019) [10]: AJCC prognostic stage groups.

TNM	Grade	Stage Group
Tis N0 M0	-	0
Tis(LAMN) N0 M0	-	0
T1 N0 M0	-	I
T2 N0 M0	-	I
T3 N0 M0	-	IIA
T4a N0 M0	-	IIB
T4b N0 M0	-	IIC
T1 N1 M0	-	IIIA
T2 N1 M0	-	IIIA
T3 N1 M0	-	IIIB
T4 N1 M0	-	IIIB
T(any) N2 M0	-	IIIC
T(any) N(any) M1a	-	IVA
T(any) N(any) M1b	G1	IVA
T(any) N(any) M1b	G2, G3, or GX	IVB
T(any) N(any) M1c	Any G	IVC

**Table 6:** The criteria for the diagnosis and staging of appendiceal neuroendocrine neoplasms, according to the *AJCC Staging Manual* (2017) [64] and *WHO Classification of Tumours of the Digestive System* (2019) [10]: definition of the primary tumor (T).

T Category	T Criteria
TX	Primary tumor cannot be assessed
T0	No evidence of primary tumor
T1	Tumor 2 cm or less in greatest dimension
T2	Tumor more than 2 cm but less than or equal to 4 cm
T3	Tumor more than 4 cm or with subserosal invasion or involvement of the mesoappendix
T4	Tumor perforates the peritoneum or directly invades other adjacent organs or structures (excluding direct mural extension to adjacent subserosa of adjacent bowel), e.g., abdominal wall and skeletal muscle

**Table 7:** The criteria for the diagnosis and staging of appendiceal neuroendocrine neoplasms, according to the *AJCC Staging Manual* (2017) [64] and *WHO Classification of Tumours of the Digestive System* (2019) [10]: definition of regional lymph node (N).

N Category	N Criteria
NX	Regional lymph nodes cannot be assessed
N0	No regional lymph node metastasis
N1	Regional lymph node metastasis

**Table 8:** The criteria for the diagnosis and staging of appendiceal neuroendocrine neoplasms, according to the *AJCC Staging Manual* (2017) [64] and *WHO Classification of Tumours of the Digestive System* (2019) [10]: definition of distant metastasis (M).

M Category	M Criteria
M0	No distant metastasis
M1	Distant metastasis
M1a	Metastasis confined to liver
M1b	Metastases in at least one extrahepatic site (e.g., lung, ovary, nonregional lymph node, peritoneum, bone)
M1c	Both hepatic and extrahepatic metastases

**Table 9:** The criteria for the diagnosis and staging of appendiceal neuroendocrine neoplasms, according to the *AJCC Staging Manual* (2017) [64] and *WHO Classification of Tumours of the Digestive System* (2019) [10]: AJCC prognostic stage groups.

TNM	Stage Group
T(X, 0) N(X, 0, 1) M1	IV
T1 N0 M0	I
T1 N1 M0	III
T1 N(X, 0, 1) M1	IV
T2 N0 M0	II
T2 N1 M0	III
T2 N(X, 0, 1) M1	IV
T3 N0 M0	II
T3 N1 M0	III
T3 N(X, 0, 1) M1	IV
T4 N0 M0	III
T4 N1 M0	III
T4 N(X, 0, 1) M1	IV

**Table 10:** Therapies used for treating inflammatory pathologies of the vermiform appendix.

Therapy	Recurrence Rate	Benefits	Limitations/Complications
Surgical resection (open appendectomy)	None [119]	Effectiveness and safety in preventing occurrence [119]	Limitations: - Complications: postoperative bleeding, wound infection, intra-abdominal abscess, and paralytic ileus, with a rate of 11.1% [120,121]
Surgical resection (laparoscopic appendectomy)	None [119]	Effectiveness and safety in preventing occurrence [119]	Limitations: - Complications: postoperative bleeding, wound infection, intra-abdominal abscess, and paralytic ileus, with a rate of 8.7% [120,121]
Antibiotic administration	High [122] 5% during the one-year follow-up period [123] 2–14% for initial antibiotic treatment, during follow-up [124] 28.6% % after an average of 4.3 years of follow-up, especially when appendicoliths are present [125] 34.0% at 2 years, 35.2% at 3 years, 37.1% at 4 years, and 39.1% at 5 years [126]	Avoiding surgical approach in most cases [122,123,126,127] Less pain, less analgesia [122] Low hospitalization rate [127] Effectiveness in about 60% of cases of simple appendicitis [123]	Limitations: applies only to uncomplicated cases [128] Complications: perforation and pelvic sepsis [126], occurring in 8.1 per 100 participants, being higher for those with appendicoliths (20.2 per 100 participants) compared to those with no appendicoliths (3.7 per 100 participants) [127] Some cases (29%) need appendectomy after 90 days [127]
Endoscopic retrograde appendicitis therapy	2.86% during the first six months of postoperative follow-up [129] 5 to 6.2% [130]	Nonoperative and minimally invasive, safe, and effective endoscopic treatment [129,131]	Limitations: applies to uncomplicated cases [131] Complications: increased risk of bowel cancer [132]

## Data Availability

Not applicable.
